# Contributions of proteomics to understanding phagosome maturation

**DOI:** 10.1111/j.1462-5822.2008.01140.x

**Published:** 2008-07

**Authors:** Lindsay D Rogers, Leonard J Foster

**Affiliations:** Cell Biology Proteomics group, Centre for High-throughput Biology and Department of Biochemistry and Molecular Biology, University of British ColumbiaVancouver, BC, Canada V6T 1Z4.

## Abstract

In metazoans macrophage cells use phagocytosis, the process of engulfing large particles, to control the spread of pathogens in the body, to clear dead or dying cells, and to aid in tissue remodelling, while the same process is also used by unicellular eukaryotes to ingest food. Phagocytosing cells essentially swallow the particles, trapping them in vacuoles called phagosomes that go through a series of maturation steps, culminating in the destruction of the internalized cargo. Because of their central role in innate immunity and their relatively simple structure (one membrane bilayer surrounding a single particle), phagosomes have been a popular subject for organelle proteomics studies. Qualitative proteomic technologies are now very sensitive so hundreds of different proteins have been identified in phagosomes from several species, revealing new properties of these intriguing compartments. More recently, quantitative proteomic approaches have also been applied, shedding new light on the dynamics and composition of maturing phagosomes. In this review we summarize the studies that have applied proteomic technologies to phagosomes and how they have changed our understanding of phagosome biology.

## Phagocytosis

Professional phagocytes of the innate immune system normally destroy invading microorganisms by engulfing them into specialized vacuoles and then conferring microbicidal and degradative capabilities upon the new compartments (Vieira *et al*., 2002; Underhill, 2005; Haas, 2007). In this process, phagocytic receptors at the cell surface bind to an invading microorganism, inducing receptor clustering, which in turn initiates phagocytosis. Non-opsonic receptors recognize and bind to specific molecules or molecular patterns on the pathogen's surface, including moieties such as mannyl or fucosyl residues, lipopolysaccharides or lipoproteins. Alternatively, host-derived ligands such as complement proteins and immunoglobulins can identify and coat foreign objects and these opsonins are then recognized by cell-based receptors, including the complement receptors and the Fc_Y_ receptors. Multiple ligands on a single foreign particle force the receptors to cluster, which initiates signalling pathways unique to the receptor–ligand pair. These cascades recruit diverse enzymes and structural components that effect extensive membrane remodelling, culminating in the engulfment of the foreign particle within the cell, encased in a membranous organelle called the phagosome.

## Phagosome maturation

Following fission of the phagosome from the plasmalemma, the classic model has the newly formed organelle fusing sequentially with (maturing into) sorting (or early) endosomes, late endosomes and finally lysosomes to acquire microbicidal and degradative capabilities. Among several proteins delivered by lysosomal fusion, Slc11a1 (Nramp1) appears to be of critical importance in macrophages' ability to effectively deal with bacteria. The mechanisms behind this are poorly understood but may involve the regulation of divalent cation concentrations in the phagolysosomal lumen (Forbes and Gros, 2001). Thus, pathogens are not killed by internalization *per se*, but rather by the extremely hostile lumen of the phagolysosome. However, the order of the fusion steps is highly specific – newly formed phagosomes only fuse with sorting endosomes, and only after this event can they fuse with late endosomes, and so forth (Vieira *et al*., 2002). Based on these observations, it is generally agreed that fusion with each compartment delivers the necessary protein machinery to allow subsequent fusion steps to occur. Work in model systems of vesicle fusion has demonstrated roles for soluble *N*-ethylmaleimide sensitive attachement protein receptor (SNARE) proteins and small GTPases of the Rab family in specifying the fusion competence of two membranes and, to this end, several members of these protein families have been shown to localize to one endosome/phagosome stage or another (Duclos *et al*., 2000; Coppolino *et al*., 2001; Harrison *et al*., 2003; Bared *et al*., 2004).

## Proteomics

Proteomics is the study of the structure, expression, localization, activity, interactions and cellular roles of all the proteins in a system (de Hoog and Mann, 2004). The degree to which we can comprehensively measure any of these parameters varies, with methods for determining expression and localization (Foster *et al*., 2006) being perhaps the most advanced. In the past, two-dimensional gel electrophoresis (2DGE) was used extensively to visualize a proteome. However, identification of more than a handful of the hundreds of spots from a 2DGE experiment has not proven very efficient, leading to nanoflow high-performance liquid chromatography/tandem mass spectrometry (LC-MS/MS) surpassing 2DGE as the method of choice for most applications. Commercially available mass spectrometers and informatic tools for interpreting mass spectra have advanced to the point where laboratories experienced in LC-MS/MS can routinely and reliably identifying hundreds or thousands of proteins in exceedingly complex mixtures (Andersen *et al*., 2002; Foster *et al*., 2003; 2006). Although not yet as widely used, methods also exist for performing quantitative LC-MS/MS experiments (Gygi *et al*., 1999; Ong *et al*., 2002; 2003).

## Proteomic characterization of biochemically enriched phagosomes

Proteomic characterizations of phagosomes was initiated over a decade ago, as Desjardins *et al*. (1994a) developed a method of isolating phagosomes containing inert, low-density particles by ultracentrifugation on a sucrose density gradient. During two initial studies (see Table 1 for a summary of all studies), 2DGE was utilized to resolve proteins from latex bead-containing phagosomes isolated from two human and mouse macrophage cell lines (U937 and J774 respectively), as well as one hamster and one rat kidney cell line (BHK and NRK respectively; Desjardins *et al*., 1994a,b). Proteins were then identified by comigration with spots from a human keratinocyte-2D gel protein database and by Western blotting. Although these studies identified only a limited set of phagosomal proteins, conservation between species was shown, and changes in spot intensities over 24 h of phagosome maturation revealed the dynamic nature of the phagosome proteome during this process. Using the same isolation approach, Garin *et al*. combined 2DGE with protein identification by mass spectrometry to characterize over 140 proteins between 0 and 24 h after phagocytosis (Garin *et al*., 2001). Proteins identified included cell surface proteins, subunits of the vacuolar ATPase, vesicular fusion and fission machinery, GTPases, hydrolases and other lysosomal proteins, endosomal and exosomal markers, several cytoskeletal and coat proteins, ER proteins and mitochondrial proteins. Furthermore, the observed sequential delivery of hydrolases to phagosomes suggested heterogeneity in phagosomes and lysosomes and multiple interactions between these compartments.

**Table 1:** Chronological summary of phagosomal proteomics studies to date.

Reference	Organism/cell type	Proteomic method	Proteins IDd/quantified^a^	Maturation time (min)^b^	Quantification^c^	Particle^d^
Desjardins *et al*. (1994a)	Mouse/macrophage	2DGE	< 10/3	0.5/0, 1/1, 1/6, 1/20 (h)	Spot intensity^e^	0.8 μm LB^f^
Desjardins *et al*. (1994b)	Human, rat, hamster, mouse/macrophage, kidney	2DGE	< 20/3	1/0, 1/1, 1/6, 1/12, 1/24 (h)	Spot intensity	0.8 μm LB
Garin *et al*. (2001)	Mouse/macrophage	2DGE, MS	> 14	0.5/0, 1/1, 1/24 (h)	Spot intensity	0.8 μm LB
Pizarro-Cerda *et al*. (2002)	Human/epithelial	2DGE, MS	unknown	30	n/a	InlA- and InlB-coated LB
Kovářová *et al*. (2002)	Mouse/macrophage	2DGE, MS	6/none	2 h/48 h	n/a	*F. tularensis*
Gotthardt *et al*. (2002)	*Dictyostelium discoideum/*amoeba	2DGE	unknown	15/15	n/a	0.8 μm LB
Okada *et al*. (2005)	*Entamoeba histolytica/*trophozoite	LC-MS/MS	85	n/a	n/a	carboxylatd LB
Marion *et al*. (2005)	*Entamoeba histolytica/trophozoite*	2DGE and LC-, MS/MS	∼500/none	15	n/a	2.8 μm MB
Burlak *et al*. (2006)	Human/neutrophil	Prefractionation, LC-MS/MS and 2DGE, MS	198	30	n/a	2.0 μm LB
Gotthardt *et al*. (2006)	*Dictyostelium discoideum/*amoeba	2DGE, MS	179/925	5/0, 15/0, 15/15, 15/45, 15/105, 15/165	Spot intensity	0.8 μm LB
Jacobs *et al*. (2006)	*Tetrahymena thermophila/*protozoan	Prefractionation, LC-MS/MS	73	n/a	n/a	2.0 μm red- fluorescing PS
Okada *et al*. (2006)	*Entamoeba histolytica/trophozoite*	LC-MS/MS	159/159	0/0, 5/30, 5/60, 5/120	Sequence coverageg	carboxylatd LB
Stuart *et al*. (2007)	*Drosophila melanogaster/* embryonic-haematocyte	Prefractionation, LC-MS/MS	617	n/a	n/a	0.8 μm LB
Rogers and Foster (2007)	mouse/macrophage	LC-MS/MS	382/382	0/0, 10/0, 10/20, 10/30, 10/45, 10/60, 10/90, 10/120	SILAC– stable isotopes^h^	0.8 μm LB
Jutras *et al*. (2007)	mouse/macrophage	prefractionation, LC-MS/MS and 2DE, MS	167/167	1/1 (h)	spot and peak intensity^i^	0.8 μm LB
Boettner *et al*. (2008)	*Entamoeba histolytica/*trophozoite	LC-MS/MS	unknown /none	0/0, 5/0, 10/0, 10/60	n/a	2.7 μm carboxylated MB

a The number of proteins identified in the study/the number of proteins quantified in the study. No denominator value indicates that no quantification was done or the number of proteins quantified was not reported.

b Age(s) of phagosomes studied in minutes unless otherwise stated. A fraction (e.g. 15/45) indicates a pulse-chase experiment (e.g. where cells were exposed to the particles for 15 min and then the particles were washed away and maturation was allowed to proceed for 45 min).

c Quantitative proteomic method used. Technical accuracy of methods: stable isotopes > peak/spot intensity > sequence coverage.

d Particle phagocytosed.

e A comparison of the intensity of corresponding spots between two 2DGE.

f LB: latex bead. Distance indicated the average diameter of beads used.

g A comparison between two samples of the fraction of the whole protein sequence observed by tandem mass spectrometry.

h Stable Isotope Labelling by amino acids in cell culture. Stable isotope methods are the most accurate quantification strategies in proteomics.

i Comparison of the peak volume in one LC-MS/MS analysis with than in another.

2DGE, 2-dimensional gel electrophoresis; MS, mass spectrometry; LC-MS/MS, liquid chromatography-coupled tandem mass spectrometry; n/a, not applicable.

Subsequently, several studies have embarked on proteomic characterizations of phagosomes isolated from diverse species. In two independent reports, Soldati *et al.* characterized phagosomes from the amoeba *Dictyostelium discoideum* using a modified version of the isolation procedure described by Desjardins *et al*. (1994b). In these studies the preparation was treated with ATP to loosen the actin meshwork surrounding newly formed phagosomes and thus improve purity (Gotthardt *et al*., 2002; 2006). They identified 179 potential phagosome proteins with a similar array of functions to those previously described. Importantly, based on changes in spot and band intensities on 2D gels and Western blots, they observed that over a maturation time of 165 min, proteins could be grouped into five major clusters: those that show a distinct peak at one time point, those that show constant abundance throughout, and those that show complex patterns and peak multiple times (Gotthardt *et al*., 2006). This suggested that linear maturation is an oversimplification and that there is either significant cross-talk between endocytic organelles or that alternative parallel pathways exist for maturing phagosomes. Nozaki and colleagues identified similar classes (early, intermediate, late and biphasic profiles) of proteins in *Entamoeba histolytica* trophozoites by LC-MS/MS (Okada *et al*., 2005; 2006) using carboxylated latex-bead containing phagosomes isolated on a sucrose gradient. Using the semi-quantitative sequence coverage parameter they were able to track the relative abundances of proteins after 0, 30, 60 and 120 min of maturation.

Recent improvements in the sensitivity and resolution of commercial mass spectrometers, as well as advances in quantitative proteomic techniques, have enabled the simultaneous identification and quantification of several hundred proteins. Following isolation of latex bead-containing phagosomes from *Drosophila* S2 cells, Stuart *et al*. identified 617 potential phagosomal proteins and added 50 to 80 secondary components by looking at the phagosomal proteins in the context of an established protein–protein interaction network (Stuart *et al*., 2007). In addition, they also used RNAi and fluorescence-based cell sorting to screen 837 genes for their role in phagocytosis of *Staphylococcus aureus* and *Escherichia coli*. This effort identified great variation in genes important for the uptake of each pathogen and was overlaid on the protein–protein interaction network to verify proteins that had a function in particle internalization. From this work, tubulin, Rab-GDI and chaperonin-containing T-complexes, as well as several components of the exocyst complex, emerged as being critically important for the internalization of bacteria. Using stable isotope labelled mouse macrophages, we have recently isolated phagosomes derived from the internalization of IgG-opsonized latex beads from a sucrose density gradient. The phagosomal proteome was investigated at high temporal resolution of (0, 10, 30, 30, 45, 60, 90 and 120 min) using stable isotope labelling by amino acids in cell culture (SILAC), which enables changes in the relative abundance of peptides to be determined over time by LC-MS/MS (Rogers and Foster, 2007). This resulted in the identification and quantification of 382 potential phagosome proteins based on dynamic profiles generated from the relative intensity of metabolically labelled and unlabelled peptides. The proteins identified were similar to those from previous studies and the quantitative profiles largely matched those previously characterized. However, consistent with data from work mentioned previously, several proteins displayed complex profiles peaking in abundance on phagosomes at several time points and supporting a more complex model of the maturation pathway, possibly involving heterogeneity between compartments and/or multiple vesicle fusion events. Recently, Jutras *et al*. also utilized quantitative proteomics to analyse regulation of the phagosome proteome by interferon-γ (Jutras *et al*., 2007), showing upregulation of most known phagosomal markers such as lysosomal hydrolases, vATPase subunits and several Rabs and SNAREs by this cytokine. Interstingly, while TLRs were also found to be upregulated on phagosomes following interferon-γ treatment, receptors involved in phagocytosis of apoptotic cells (LRP/CD91) and nutrient uptake (transferring receptor) were downregulated, suggesting modulation of phagocytic cargo during an immune response.

Several studies addressing phagosomal proteomes in a range of organisms have now established a large degree of conservation within the pathway and identified a complex array of proteins as potentially or known to be associated with maturing phagosomes (Table 1). However, while several of these have validated the presence of a select few proteins on phagosomes, differentiating between real phagosome players and contaminants is still an important issue. While it is not practical to verify each protein individually, alternative methods of isolating phagosomes should prove helpful in deciphering both false positives and false negatives. With the exception of magnetic isolation of phagosomes from *E. histolytica* (Marion *et al*., 2005; Boettner *et al*., 2008), phagosomes from human neutrophils isolated on a continuous density gradient (Burlak *et al*., 2006), and the initial removal of the actin meshwork surrounding phagosomes in *D. discoideum* (Gotthardt *et al*., 2002; 2006), phagosome proteomics studies have all used the original enrichment procedure involving the isolation of low density latex beads on a sucrose density gradient.

## Sources of phagosomal membrane

The surface area of a phagocytosing macrophage remains static, or if anything, it increases despite using an area of membrane equal to or greater than its surface area to engulf particles (Hackam *et al*., 1998; Holevinsky and Nelson, 1998), suggesting that intracellular membrane source(s) is required to replenish the PM and/or to actually form the phagosome. In addition, during cross-presentation of antigens by phagocytic cells, the method of transporting antigenic peptides from the phagosomal lumen to the cytosol for subsequent transport into the ER, secretion and presentation on MHC Class I molecules is poorly defined (Huang *et al*., 1996; Rodriguez *et al*., 1999).

Of all the proteomics-based discoveries addressing phagosome biology, the reports by Desjardins and colleagues that the endoplasmic reticulum (ER) is directly involved in phagocytosis (Garin *et al*., 2001; Gagnon *et al*., 2002) have had the largest impact. Their work, and that of others (Guermonprez *et al*., 2003; Houde *et al*., 2003), presented a very attractive explanation for how antigens from intracellular pathogens can be transported from phagosomes for cross-presentation, and proposed the ER to directly contribute the vast majority of membranes used to construct the forming phagosome. More recently however, through extensive biochemical assays, fluorescent imaging, and electrom microscopy-based experiments, most of the lines of evidence in favour of ER-mediated phagocytosis have been challenged (Touret *et al*., 2005a,b). In addition to the plasmalemma, endosomal and post-Golgi membranes (Desjardins *et al*., 1994a; Jahraus *et al*., 1998), as well as exosomal membranes, have been shown to supply membrane to the forming phagosomal cup (Lee *et al*., 2007).

**Fig. 1:**
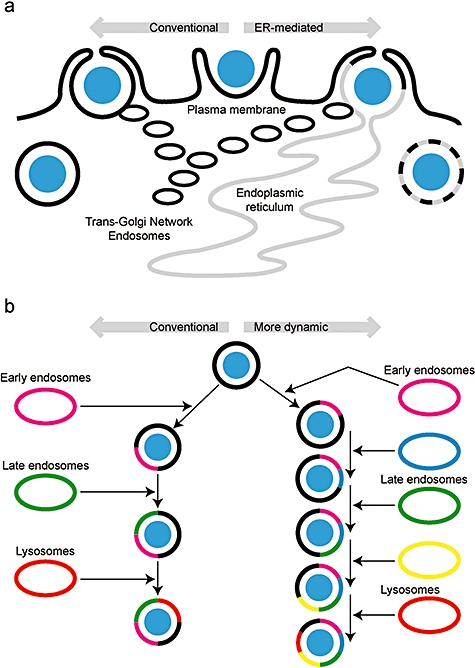
Models of phagosome formation and maturation. A. In the conventional model of phagocytosis (left side) the plasma membrane is the major source of membrane and the composition of the very early phagosome should be very similar to the plasma membrane. In the ER-mediated model (right side of figure) a large fraction of the phagosomal membrane is contributed directly from the ER. A significant prediction of this model is that the very early phagosome should contain a significant fraction of ER membrane. In both models other endomembranes, including the *trans*-Golgi network, are also likely to contribute membranes. B. In the conventional model of phagosome maturation (left side) the phagosome fuses sequentially with the early endosome, late endosome and lysosome. Two quantitative proteomic studies (Gotthardt *et al*., 2006; Rogers and Foster, 2007) have demonstrated that there are likely more distinct fusion events, presumably with subpopulations of the three main classes of endosomes.

Many groups, including those using proteomics, have consistently reported ER markers on phagosome preparations (Garin *et al*., 2001; Gagnon *et al*., 2002; Touret *et al*., 2005a; Gotthardt *et al*., 2006; Rogers and Foster, 2007; Stuart *et al*., 2007), but until recently these reports were only qualitative. The ER-mediated model of phagocytosis (Fig. 1) predicts that a very significant fraction of the phagosome membrane should be derived from the ER. No numbers are specified in the proposed model by Gagnon *et al.* but from the cartoon models based on electron microscopy images approximately 50% of the phagosomal membrane may come from the ER, with the remaining originating from the plasma membrane (PM; Gagnon *et al*., 2002). On the other hand, in the opposing model where the ER does not comprise the majority of forming phagosomes, the fraction of the phagosome comprised of ER should be very small (Fig. 1). By fluorescent imaging Touret *et al*. have also shown 50% of GPI-YFP to be retained on the phagosomal membrane 5 min after particle internalization, suggesting that half the phagosomal membrane is comprised of plasmalemma at this time (Touret *et al*., 2005b). These observations and models then suggest a testable hypothesis: if the ER-mediated model is correct then the amount of PM on the phagosome, as a fraction of the total PM, should be about equal to the fraction of ER on the phagosome, after adjusting for the total surface areas of the PM and the ER. In a recent study, quantitative proteomics was used to use relative protein levels to estimate the percentage PM and ER membranes on phagosomes 10 min after internalization yielding approximately 10% and 0.2%, respectively, a ratio of 50:1 (Rogers and Foster, 2007). Thus, if there were 50 times more ER membrane than PM in a macrophage these data would support the ER-mediated phagocytosis model. However, the ER:PM ratio is probably closer to 2:1 (Blouin *et al*., 1977), suggesting that the contribution of the ER in phagocytosis is negligible.

There is little doubt, however, that some ER proteins are found on phagosomes (Gagnon *et al*., 2002; Touret *et al*., 2005a; Rogers and Foster, 2007). While not comprising a sizable faction of the phagosomal membrane, it has been elegantly shown that these proteins function to modify and export phagosomal cargo into the cytosol for subsequent uptake in the ER and presentation by MHC Class I molecules (Guermonprez *et al*., 2003; Houde *et al*., 2003; Ackerman *et al*., 2006). While not yet addressing this issue directly, data from proteomic studies do support the cross-presentation competency of phagosomes, having identified MHC Class I molecules, components of the MHC class I loading complex, and proteasome subunits on phagosome preparations (Jutras and Desjardins, 2005; Burlak *et al*., 2006).

Proteomics reports have also consistently identified mitochondrial proteins in phagosomal preparations (Garin *et al*., 2001; Burlak *et al*., 2006; Gotthardt *et al*., 2006; Okada *et al*., 2006; Rogers and Foster, 2007). Thus, small amounts of a select class of mitochondrial proteins may also comprise a portion of the phagosomal membrane, possibly to promote oxidative potential (Burlak *et al*., 2006; Okada *et al*., 2006). While these proteins may be delivered indirectly through other compartments (Neuspiel *et al*., 2008), Burlak and colleagues have confirmed the presence of some of these players by immunofluorescence microscopy (Burlak *et al*., 2006).

## The Holy Grail for phagosome proteomics: bacteria-containing vacuoles

Opsonized latex beads are an excellent tool for exploring normal phagosome maturation; they are inert, easily manipulated and can be obtained in different sizes to mimic various targets a phagocyte is likely to meet. However, some of the most interesting aspects of phagosome maturation revolve around the abilities of intracellular pathogens to bypass the normal maturation process. Two examples of many such mechanisms are seen in *Salmonella enterica*, which prevents formation of the phagolysosome (Haraga *et al*., 2008), and *Mycobacterium tuberculosis*, which blocks lumenal acidification by preventing conversion of Rab proteins between their GDP- and GTP-bound states (Deretic *et al*., 2006). Several groups have reported procedures for biochemical enrichment of bacteria-containing vacuoles (BCV) but no proteomic analysis of such a compartment has been reported (Kovářová *et al*., 2002 analysed *Francisella tularensis*-containing phagosomes by 2DGE but only reported the identities of two host-derived proteins). We, and probably others, have expended significant effort to try to analyse the proteome of BCV, those containing *S. enterica* serovar Typhimurium in our case. The conventional sucrose density gradient approach has been unsuccessful because BCV are a very similar density to other compartments of the cell, most significantly mitochondria that probably share a common ancestry with bacteria (Margulis, 1968) and would thus have a similar density. Attempts to shift the density of BCVs by using latex nanoparticles or by making the BCVs magnetic by phagocytosing magnetic beads together with bacteria were not clean enough for analysis, and fluorescence-activated cell sorting of BCVs containing green fluorescent protein-expressing *S. typhimurium* was not quick enough to obtain sufficient material of the unstable BCV. Efforts to alter the density of mitochondria or immunodeplete them in the hopes of cleaning up that region of the gradient were successful in that the mitochondrial contamination was decreased but unfortunately this only revealed the presence of many other host-derived membranous compartments in higher abundance that the BCVs. Nonetheless, as current work unveils a vast array of different BCVs comprising few to several bacteria and varying greatly in size and likely also in content (Birmingham *et al*., 2008), the notion of purifying these vacuoles to analyse their composition and various functionalities becomes increasingly intriguing (Birmingham *et al*., 2008).

## Conclusions

Global, unbiased proteomics approaches have made significant contributions to phagosome biology, perhaps more so than any other area of cell biology. Proteomics opened up the debate over the role of the ER in phagocytosis (Garin *et al*., 2001) and more advanced quantitative methods can now be used to test hypotheses arising from this model (Rogers and Foster, 2007). A very comprehensive systems biology approach to studying phagosomes demonstrated a role for the exocyst complex in phagosome maturation (Stuart *et al*., 2007). The use of more advanced quantitative techniques to evaluate the maturing phagosomal proteome revealed a role for heterotrimeric G-proteins in phagosme maturation (Gotthardt *et al*., 2006) and has suggested that the classical model of three consecutive fusions events with different endosomal systems is probably overly simplistic (Gotthardt *et al*., 2006; Rogers and Foster, 2007).

Phagosome proteomics studies typically use opsonized latex beads to model phagocytosis (for exception see Kovářová *et al*., 2002) and for the normal process this is probably reasonable. However, one of the most interesting questions about phagocytosis is how certain intracellular pathogens are able to manipulate normal phagosome maturation in order to survive inside the host (e.g. *S. enterica*, *M. tuberculosis*). Thus, while there are some significant challenges yet to be addressed in the biochemistry, we feel that the future of phagosome proteomics is in exploring how such pathogens alter the composition of the phagosomal membrane.
